# The COP9 signalosome variants CSN^CSN7A^ and CSN^CSN7B^ form complexes with specific CRLs which are targets of autophagy

**DOI:** 10.1042/EBC20253018

**Published:** 2025-08-26

**Authors:** Wolfgang Dubiel, Dawadschargal Dubiel

**Affiliations:** Institute of Experimental Internal Medicine, Medical Faculty, Otto von Guericke University, Magdeburg, Germany

**Keywords:** adipogenesis, autophagy, cell cycle inhibitor p27^KIP^, COP9 signalosome (CSN), cullin-RING-ubiquitin ligases (CRLs), RAB18

## Abstract

COP9 signalosome (CSN) is a representative of the ZOMES complexes, which further consist of the 26S proteasome LID and the eukaryotic translation initiation factor 3 (eIF3), key players in proteostasis. Whereas the eIF3 complex has a role in general translation initiation, the LID, as part of the regulatory particle of the 26S proteasome, is the main cellular proteolytic machinery, specifically degrades ubiquitylated substrates. Interestingly, CSN is associated with the production of ubiquitylated substrates. Contrary to its paralogous complexes, CSN appears as variants. Our interest mainly lies in the variants CSN^CSN7A^ and CSN^CSN7B^. The CSN variants form stable complexes with cullin-RING-ubiquitin ligases (CRLs). While CSN^CSN7A^ preferentially interacts with CRL3, CSN^CSN7B^ binds to CRL4A. CSN^CSN7A^-CRL3 and CSN^CSN7B^-CRL4A complexes are stored in human cells as latent complexes. During adipogenesis in LiSa-2 model preadipocytes, the complexes are integrated into different functions. CSN^CSN7A^-CRL3 complexes are recruited by rat sarcoma-related small GTPase 18 to the membrane of lipid droplets, where they are neddylated. CSN^CSN7B^-CRL4A complexes lose their substrate receptor and stop degrading p27, causing cell cycle arrest necessary for adipogenesis. The C-terminal approximately 60 amino acids of CSN7A and CSN7B are essential for the specific binding to CRLs. Without C-termini, CSN^CSN7A1-200^ and CSN^CSN7B1-200^ lose CRL3 or CRL4A and their function. CSN-CRL complexes are degraded by a selective macroautophagic pathway. In the presence of the specific inhibitor CSN5i-3, the appearance of ubiquitylated CSN-CRL complexes was detected in cells. Nonfunctioning CSN-CRL particles are fixed as cargo before forming vesicles as autophagosomes following degradation via lysosome.

## Introduction

Mammalian COP9 signalosome (CSN) has eight core subunits (CSN1–CSN8). It possesses six proteasome LID-CSN-initiation factor 3 domain subunits (CSN1–CSN4, CSN7, and CSN8) and two MPR1/PAD1 N-terminal (MPN) domain subunits (CSN5 and CSN6) [1]. The CSN is conserved from yeast to *mammalia*. The plant CSN—discovery, conservation, activity, and function—was described in detail by Deng and colleagues [2] and will not be further considered here. CSN is a major ZOMES complex. Like its paralogous complexes, the 26S proteasome LID and the eukaryotic translation initiation factor 3 (eIF3), CSN is characterized by a metallodeubiquitylating module, which represents a heterodimer consisting of one MPN+domain (CSN5) and one ‘non-active’ MPN domain subunit (CSN6) [3]. The eIF3, LID, and CSN participate in the regulation of the balance of the functional proteome. The eIF3 complex plays a role in general translation initiation [4]. On the other hand, the LID, as part of the regulatory particle of the 26S proteasome [5], is one of the major proteolytic machineries in cells and specifically degrades ubiquitylated substrates [6]. The CSNs stably interacting with about 200 cullin-RING-ubiquitin ligases (CRLs) [1] ubiquitylate a large variety of substrates for degradation [7]. In *mammalia,* cullins 1–9 (CUL1–CUL9) are the backbone of CRLs, which further consist of a RING-domain protein, E2 proteins, and a substrate receptor (SR) [7,8]. In the CSN, the metallodeubiquitylating module has a deneddylating activity that controls CRLs. Specifically, it removes NEDD8 (Neural precursor cell-Expressed Developmentally downregulated 8) from cullins and thus regulates the ubiquitylating activity of CRLs [9]. In this review, we summarize recent findings on special functions of CSN variants as CSN-CRLs complexes during adipogenesis and their degradation by autophagy. Like single-cellular proteins, huge protein complexes such as ZOMES (CSN, LID, and eIF3) exist in cells in a dynamic state, a balance between synthesis and degradation, which changes depending on functional requirements. While the degradation of the 26S proteasome complex by autophagy has been described in recent years, the destiny of CSN-CRL complexes remained obscure. Here, we will compile our recent data on CSN-CRL degradation via autophagy. We will compare it with 26S proteasome autophagy during different autophagic steps.

## Diversity of the COP9 signalosome

**Figure 1:**
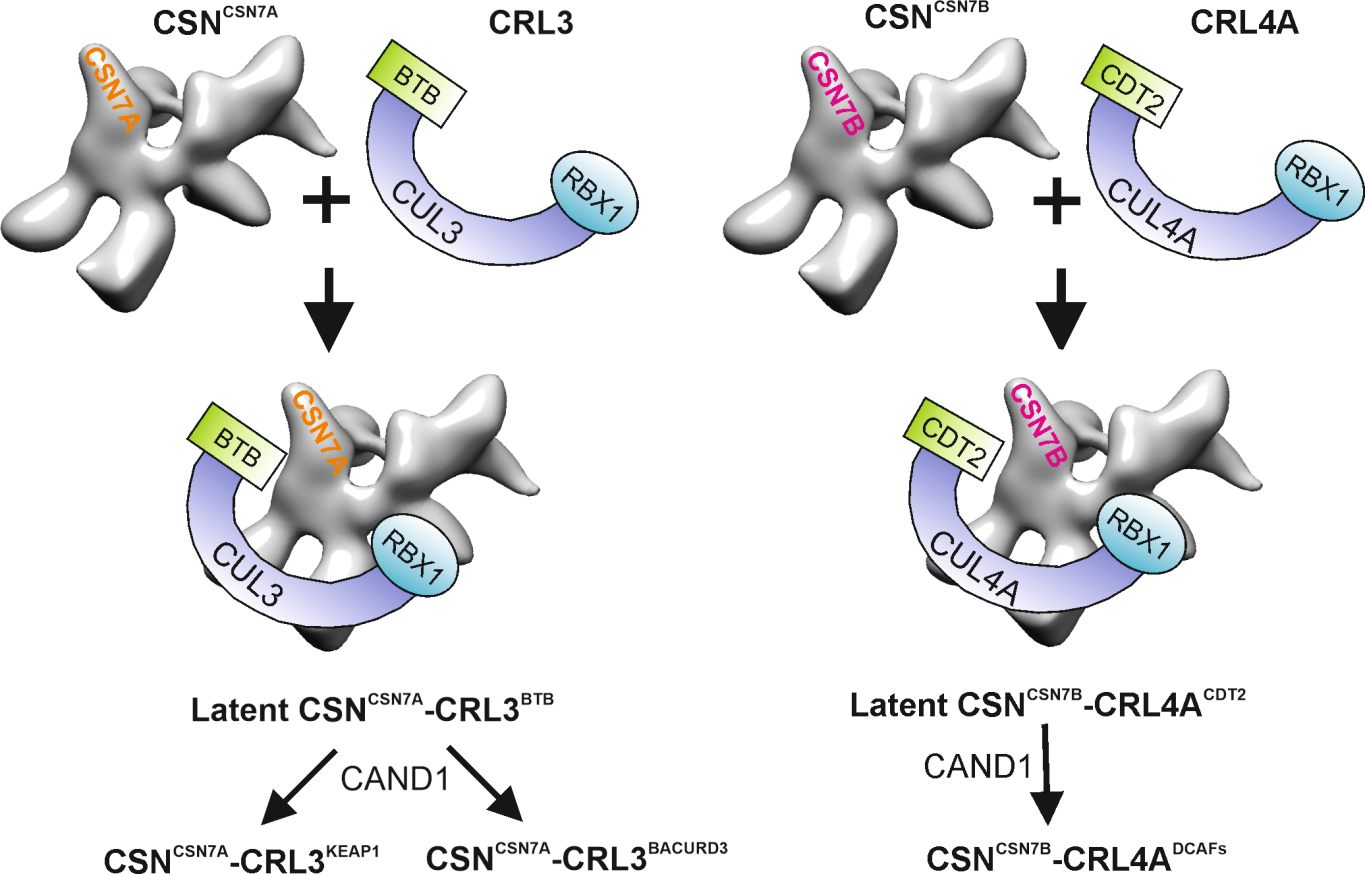
Preferential binding of CSN variants to CRLs. The CSN variant CSN^CSN7A^ preferentially binds to CRL3 and the CSN variant CSN^CSN7B^ to CRL4A. During adipogenesis CSN^CSN7A^-CRL3^BTB^ [bric-a-brac, tramtrack, and broad complex (BTB)/Pox virus and Zinc finger protein] substrate receptor (SR) is exchanged for KEAP1 and BACURD3 with the help of CAND1. CSN^CSN7B^-CRL4A^CDT2^ loses its SR CDT2 (Cdc10-dependent transcript 2) to another DCAFs (DCAF – DDB1 and CUL4-associated factors) protein. CAND1, cullin-associated and neddylation-dissociated 1; CRL, cullin-RING-ubiquitin ligase; CSN, COP9 signalosome; CUL, cullins; RBX1, RING-box protein1.

The CSN is more heterogeneous than indicated by the structure of the eight-subunit complex [10]. A fraction of the cellular CSN contains a noncanonical subunit called CSN acidic protein) [11]. In addition, CSN interacts with deubiquitylating enzymes (DUBs). The CSN itself is a DUB, which deneddylates cullins and reacts with other DUBs. Recently, we published that the CSN is a multi-DUB complex [12]. For example, the ubiquitin-specific protease 15 (USP15) binds to CSN7A and the ubiquitin-specific protease 48 (USP48) to CSN7B, forming the complexes CSN^CSN7A-USP15^ and CSN^CSN7B-USP48^ [1]. USP15 is a DUB that regulates mitophagy [13], whereas USP48 is mostly localized to the nucleus [14]. In addition, the CSN binds to several proteins, including rat sarcoma-related small GTPase 18 (RAB18) or caveolin 1 [1]. High diversity of the CSN is due to the presence of paralogous subunits. While plant CSN has CSN5A and CSN5B, as well as CSN6A and CSN6B [15], there are CSN7A and CSN7B, as well as CSN8A and CSN8B, in mammalian cells [1]. All paralogs form their own CSN complexes called variants. We focused on the study of CSN^CSN7A^ and CSN^CSN7B^ variants. In *mammalia*, the CSN^CSN7A^ and CSN^CSN7B^ variants co-exist, performing a wide variety of functions. This has to do with the most important CSN interaction partner, the CRLs. When human cells are mildly lysed (with single-detergent buffer), stable latent CSN-CRL complexes can be found (Figure 1). Latent CSN-CRL complexes are not active but can be quickly activated—most likely by substrates. A closer analysis shows that CSN^CSN7A^ interacts preferentially with CRL3 and CSN^CSN7B^ with CRL4A/B [1]. These complexes are indestructible by the neddylation inhibitor MLN4924 [16] and by the deneddylation inhibitor of CSN, CSN5i-3 [17]. They are available for cell functions in the steady state [18]. In the current model, CRL dynamics are regulated by cycles of deneddylation and cullin-associated and neddylation-dissociated 1 (CAND1) to adapt CRLs to changes of substrate availability [19]. Based on new data, we suggest that both models exist. Latent CSN-CRLs are already present under steady-state conditions and serve as the main reservoir available in most cells for diverse cell functions [1]. However, under specific conditions such as cell differentiation, a fast exchange of SRs requires CAND1 cycles (Figure 1). To study the functions of CSN-CRL complexes in cell differentiation, we used the model of adipogenesis.

## Functions of CSN^CSN7A^-CRL3 and CSN^CSN7B^-CRL4A during adipogenesis

Adipogenesis is the differentiation of preadipocytes into mature adipocytes. In LiSa-2 preadipocytes, differentiation of mature LiSa-2 cells takes about 14/15 days. Adipogenesis is initialized by a hormone mixture (HM) consisting of 1 nM insulin, 20 pM triiodothyronine, and 1 mM cortisol. Upon HM, LiSa-2 preadipocytes pass through different phases of differentiation: mitotic clonal expansion (MCE) (1–2 days), post-mitotic growth arrest (PGA) (3–8 days), and terminal differentiation (TAD) (9–15 days) [20]. During adipogenesis, lipid production can be detected by Oil Red-O staining. Although in MCE no lipids are detected, in PGA and especially in TAD, produced lipids accumulate in lipid droplets (LDs). MCE is characterized by one or two cell divisions in LiSa-2 cells. During that time, the key regulators of adipogenesis, such as peroxisome proliferator-activated receptor γ and leucine zipper CCAAT-enhancer binding protein α, are expressed and execute their tasks during adipogenesis.

What is the function of CSN-CRL complexes in adipogenesis? To find out, we tried to knockout *CSN7A* and *CSN7B* in LiSa-2 cells. Unfortunately, in LiSa-2 cells, the knockout of *CSN7A* was lethal. Therefore, we used a knockdown of *CSN7A*. In contrast, the knockout of *CSN7B* was not lethal in LiSa-2 cells. Both the knockdown of *CSN7A* and the knockout of *CSN7B* led to inhibition of lipid accumulation in LDs and to the blockage of adipogenesis. Under normal conditions, at the beginning of MCE, the cyclin-dependent kinase (CDK) inhibitor p27 accumulates—a hallmark of adipogenesis in LiSa-2 cells [1]. One reason is that CSN-CRL1^SKP2^ loses its SKP2 via CAND1 and does not ubiquitylate p27 anymore for degradation with the 26S proteasome. CAND1 is necessary to detach SKP2 from the complex [21]. CSN^CSN7B^-CRL4A^CDT2^ is another enzyme involved in the degradation of p27. It loses its SR during the first hours of MCE [1] and contributes to the accumulation of p27 in the cytosol and the nucleus at the end of MCE. Most likely, it binds to another DCAF (DDB1- and CUL4-associated factors) protein (Figure 1) [22]. We interpreted the effect of *CSN7B* knockout followed by the loss of CSN^CSN7B^ complexes as follows. The loss of CSN^CSN7B^ complexes leads to uncontrolled degradation of p27 by CRL4A, which prevents the accumulation of p27 in MCE [20] and blocks adipogenesis. During an early phase of adipogenesis, CSN^CSN7A^-CRL3^BTB^ [bric-a-brac, tramtrack, and broad complex (BTB)/Pox virus and zinc finger protein], CUL3 obtains the SR KEAP1 in a CAND1-dependent reaction. Some BTB proteins are exchanged for KEAP1, which specifically ubiquitylates the adipogenesis inhibitor C/EBP homologous protein (Figure 1) [23]. The SR exchange factor CAND1 is also necessary for the installation of BACURD3 in the CSN^CSN7A^-CRL3^BACURD3^ to degrade RHOA for the remodeling of the cytoskeleton (Figure 1) [24].

The knockdown of CSN7A, with the consequence of the loss of the CSN^CSN7A^ variant, conveyed a completely different function. By confocal fluorescence microscopy, we found that, under normal conditions, CSN^CSN7A^-CRL3 is recruited by RAB18 to LD membranes. CSN7A, CUL3, and RAB18 can be detected simultaneously on the LD membrane [20]. If CSN7A is absent, there are no LDs. Unfortunately, the correct SRs of the CSN^CSN7A^-CRL3 complexes on the LDs are not yet known. Therefore, the function of CSN^CSN7A^-CRL3 on LDs can only be speculated. On the other hand, we know that CSN^CSN7A^-CRL3 is activated on LD membranes, meaning it is neddylated [24].

## CSN7A- and CSN7B-C-termini are responsible for binding to CRLs

We cut off the C-terminal amino acids of CSN7A (amino acids 201–275) and CSN7B (amino acids 201–273). The truncated FLAG-tagged DNA constructs FLAG-CSN7A^1-200^ and FLAG-CSN7B^1-200^ were stably transfected into LiSa-2 cells. FLAG-CSN7A^1-200^ and FLAG-CSN7B^1-200^ were integrated into endogenous complexes. Under these conditions, the CSN^CSN7A1-200^-CRL3 and the CSN^CSN7B1-200^-CRL4A complexes fall apart [1]. In both cases, elimination of the C-termini of CSN7A and CSN7B leads to a block of adipogenesis. There is a special human disease condition where the bond between CSN and CRL is disturbed. The so-called Gordon syndrome is associated with hyperkalemia, hypertension, and obesity. As described by Schumacher et al. [25], the deletion of exon 9, amino acids 405–459 of CUL3, leads to the loss of binding to CSN. CUL3 is hyperneddylated but unable to ubiquitylate its substrates—the WNK kinases. It makes self-ubiquitylation of the CRL3 leading to degradation.

## CSN degradation by autophagy

### General on autophagy

**Figure 2:**
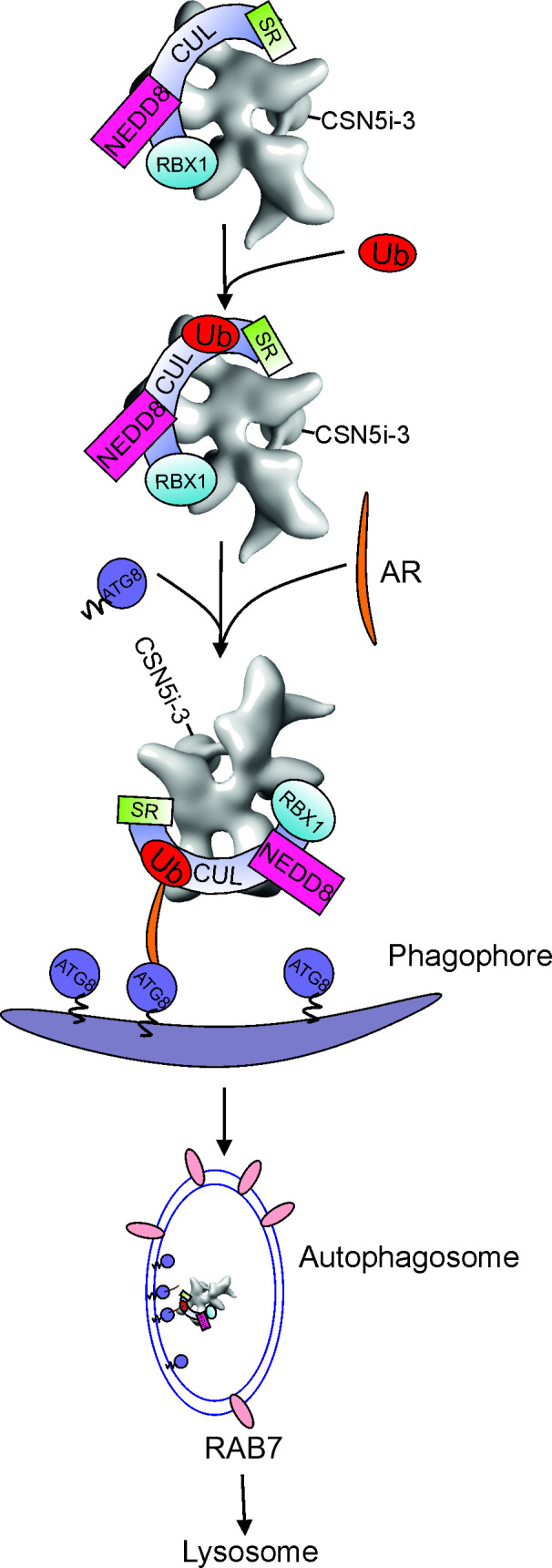
CSN-CRL complexes are degraded by selective macroautophagy. In the presence of the specific CSN inhibitor CSN5i-3, CSN-CRL complexes are ubiquitylated probably on cullins via self-ubiquitylation. With the help of lipidated ATG8 and autophagic receptor (AR), the inhibited CSN-CRL complex binds to the phagophore. This is followed by the formation of the autophagosome, the fusion with the lysosome and subsequent degradation via lysosomal proteases. RAB7 is involved in the fusion process of autophagosomes with lysosomes. ATG, autophagy; CSN, COP9 signalosome; CUL, cullins; SR, substrate receptor.

Degradation of the CSN had remained unclear until recently. Its homologue LID, a subcomplex of the 26S proteasome [26], is degraded as a whole complex and has a half-life of 16 h in fibroblasts [27] and over two weeks in rat liver cells [28]. However, under specific conditions, 26S proteasome degradation can be rapid and extensive. All cellular organisms require mechanisms to eliminate dysfunctional single proteins or protein complexes. In eukaryotes, two degradation pathways, the ubiquitin-proteasome system (UPS) and autophagy, balance proteomic homeostasis. The UPS degrades short-lived or misfolded soluble single proteins upon smuggling into the cavity of proteasomes [29,30]. In comparison, autophagy eliminates larger protein complexes or organelles or pathogens with big sizes exceeding the gate-opening capacity of proteasomes [31–33]. Proteasome and autophagy deficiencies lead to diseases [34]. The most common autophagic pathway for the degradation of protein complexes is macroautophagy. The process is accomplished by autophagy-related proteins (ATG proteins). The characteristic of macroautophagy features the formation of phagophore membranes from endoplasmic reticulum followed by a set of processes [35]. The result is the formation of small vesicles called autophagosomes filled with cargos, followed by degradation via lysosomes (Figure 2). Thereby, the Ub (ubiquitin)-like protein ATG8 is conjugated to the inside of autophagosome membrane via lipidation. Lipidated ATG8 has two functions. One is for membrane expansion and autophagosome closure. The other is to attach cargo to the enveloping membrane through interactions between ATG8 and autophagic receptors that recognize specific cargo (Figure 2) [32,35–37]. Before the closure of the membrane, large macromolecules bind to ATG8. Then, autophagosomes with cargos are fused with lysosomes following the contents of vesicles that are consumed by lysosomal proteases. Autophagosomes are loaded with RAB7, which is involved in this fusion process [38].

### Degradation of the LID together with the 26S proteasome by autophagy

In humans and plants, it has been confirmed that proteasomes are degraded by both bulk and starvation-induced autophagy [39,40]. Additionally, LID is degraded as a component of the 26S proteasome complex via different macroautophagy types upon starvation and treatment with specific proteasome inhibitors called proteaphagy [31,32,41]. Both cytoplasmic and nuclear 26S proteasomes are substrates of autophagy. Nuclear 26S proteasomes are degraded via macroautophagy after being expelled from the nucleus. In *mammalia* cells, upon amino acid starvation, the proteasome is translocated from the nucleus to the cytoplasm as a complete complex [42]. In contrast, yeast 26S proteasomes must be dissociated first into core particles and regulatory particles before export [43]. Ubiquitylation of 26S proteasome subunits attracts non-functioning complexes for autophagic clearance. Ubiquitylation is a key regulatory event in quality control in UPS and autophagy. As compared with the UPS, it is very rarely known about E3 ligases of autophagy in cells. In yeast, three E3 ligases sequentially ubiquitylate proteasomes for autophagy [41]. Specific receptors such as p62, RPN10 (26S proteasome regulatory subunit 10), and CUE5 (coupling of Ubiquitin conjugation to endoplasmic reticulum (ER) protein 5) recognize one side of the ubiquitin molecules on the affected proteasome subunits and the phagophore membrane hanging ATG8 molecules on the other side [31,44,45]. As compared with LID complex, autophagic clearance of CSN-CRL complexes had remained obscure.

### The CSN is degraded by autophagy together with its CRL

Our recent data confirm that the CSN-CRL complex is degraded by macroautophagy upon serum starvation and inhibition by CSN5i-3 in human cells [46]. The starvation-induced degradation of CSN-CRL complexes, like 26S proteasomes, serves as a free amino acid reservoir to increase starving cell survival [47]. CSN-CRL complexes occur in the nucleus as well as in the cytoplasm, whereas autophagic machinery is located exclusively in the cytoplasm. Details on the export of non-functioning CSN-CRL complexes remain obscure. In *yeast*, specific ubiquitylation is necessary to expel the 26S proteasome complex from the nucleus [41]. We have shown that in human cells, specifically, CSN5i-3-inhibited CSN-CRL complexes have been quickly routed via the secondary selective macroautophagic pathway (see Figure 2) [46]. The selectivity of macroautophagy is determined by ubiquitylation. Only in the presence of the specific CSN inhibitor CSN5i-3 [17], the production of ubiquitylated CSN-CRL complexes was detected in cells. Our subsequent studies identified cullins as target subunits and as triggers for Ub-modification of CSN-CRL complexes (see Figure 2). Therefore, we call the process self-ubiquitylation. In the case of mammalian proteasomes, certain proteasome subunits are ubiquitylated [44]. Very likely, ubiquitylated CSN-CRL complexes bind to specific autophagic receptors, bridging to ATG8 inside of phagophore membranes (Figure 2). Thus, non-functioning CSN-CRL particles are fixed as cargo before forming vesicles as autophagosomes following degradation via the lysosome (Figure 2).

Moreover, CSN-CRL mutations, like the loss of CSN7A- or CSN7B-C-termini, relate to the decay of the complexes [1], are very unstable in cells, and are mostly localized in cytoplasm. Presumably, CSN-CRL mutations induce autophagic degradation of CSN and CRL as individual forms. Further studies are certainly required to fully understand the degradation of CSN complexes via autophagy.

## Conclusions

The functions of CSN^CSN7A^-CRL3 and CSN^CSN7B^-CRL4B during adipogenesis in LiSa-2 cells are completely different. While CSN^CSN7A^-CRL3 acts on the membranes of LDs, CSN^CSN7B^-CRL4A gives off its SR CDT2 and is involved in the accumulation of the cell cycle inhibitor p27 in the early MCE phase. Detailed studies revealed that the C-termini of CSN7A and CSN7B are important for binding to CRLs. The loss of amino acids 201–275 of CSN7A and of 201–273 of CSN7B leads to the decay of the CSN-CRL complexes and loss of their activity. CSN-CRL complexes are degraded by autophagy. However, there are still unknown details about the degradation of CSN and CSN-CRL complexes in human cells. The following questions remain obscure: (i) which specific autophagic receptors (AR) are responsible for targeted degradation of CSN or CSN-CRL complexes via autophagy? (ii) Is there any dysregulation on the clearance of mutated or nonmutated CSN complexes during human diseases, e.g. cancer-initiated carcinogenesis? (iii) Are there any pathogenic situations that influence the degradation of CSN, creating advantages for themselves? (iv) Can the specific molecular mechanisms of CSN autophagic degradation offer multiple targets for the drug development of different human diseases? Further investigations of the CSN-CRL complex functions and degradation are necessary to understand their exact role and to develop appropriate drugs.

**Summary**

- The CSN^CSN7A^ variant preferentially reacts with cullin-RING-ubiquitin ligase 3 (CRL3), and CSN^CSN7B^ binds to CRL4A.
- In adipogenesis, CSN^CSN7A^-CRL3 and CSN^CSN7B^-CRL4A have different essential functions. While CSN^CSN7A^-CRL3 controls the functions of lipid droplets, CSN^CSN7B^-CRL4A is responsible for the accumulation of p27.
- CSN-CRL complexes are degraded via selective macroautophagy.
- The role of CSN-CRL complexes in the regulation of cellular processes and in diseases is generally underestimated.

## Open Access

This article has been published open access under our Subscribe to Open programme, made possible through the support of our subscribing institutions, learn more here: https://portlandpress.com/pages/open_access_options_and_prices#conditional

## Abbreviations

ATG proteins: autophagy-related proteins
BTB: bric-a-brac, tramtrack, and broad complex
CAND1: cullin-associated and neddylation-dissociated 1
CRLs: cullin-RING-ubiquitin ligases
CSN: COP9 signalosome
CUL: cullins
DUBs: deubiquitylating enzymes
HM: hormone mixture
LDs: lipid droplets
MCE: mitotic clonal expansion
MPN: MPR1/PAD1 N-terminal
PGA: post-mitotic growth arrest
RAB18: rat sarcoma-related small GTPase 18
SR: substrate receptor
TAD: terminal differentiation
UPS: ubiquitin-proteasome system
USP15: ubiquitin-specific protease 15
USP48: ubiquitin-specific protease 48
eIF3: eukaryotic translation initiation factor 3
